# West Chicago Medical Society

**Published:** 1881-06

**Authors:** 


### ARTICLE VI.

### WEST CHICAGO MEDICAL SOCIETY.

Stated meeting, May 9. Dr. Norman Bridge, President, in the chair.

Mr. S. C. Stanton read an abstract of a paper on

#### NERVE STRETCHING, by Drs. Lee and Fenger.

The operation had been first performed by Billroth, unintentionally, within the last decade, but Dr. von Nussbaum, of Munich, was the first to perform it for the relief of pain. It had now been tried all over the world, and in the following diseases: Idiopathic sciatica, secondary sciatica, neuralgia of the fifth pair, mimic spasms, neuralgia of the accessory nerve of Willis, nervous spasms of the extremities, epilepsy, traumatic paralysis, tetanus, and locomotor ataxia. Eight cases of nerve stretching in idiopathic sciatica were on record, almost all successful. The force exercised must be sufficient to cause a sensation of something giving way in the nerve. The force calculated to break the sciatic nerve was 130 pounds. In seven cases of secondary sciatica with disease of the spinal cord, the operation had failed in most of them.

Six successful cases in nine of neuralgia of the fifth pair had been recorded. The supra-orbital, the infra-orbital, and the inferior dental had been stretched. In eleven cases of neuralgia following injuries to various nerves, eight cures had resulted. Esmarch successfully stretched the nerves for the cure of neuralgia in stumps of amputated limbs. In five cases of mimic spasm, in which the nerve was stretched, the trouble disappeared. In epilepsy, paralysis, and tetanus, the results were not very satisfactory. Of twenty-one cases of tetanus operated on, nine had recovered, and twelve died. Dr. Fenger remarked that a greater number of the successful cases had been recorded than of the failures; and we should not overlook the fact that in tetanus remedies had been administered also.

Nerve stretching should be done with antiseptic precautions, but the setting in of erysipelas, and the sloughing of the wound, which happened in a few cases, did not prevent the relief of pain in the nerves stretched. Taking everything into consideration, this operation is safer and more satisfactory than section or amputation of the nerves.

In some doubtful cases of locomotor ataxia, with pain, the latter had been relieved, and co-ordination returned. In one of these, Esmarch stretched the axillary nerves, and the locomotor ataxia disappeared. Only the painful nerves should be stretched. Two cases of anæsthetic leprosy, in seven, had been benefited. The modus operandi of that new surgical measure is very obscure, for a want of experiments on the lower animals.

As these various cases showed, the operation had given favorable results in a variety of wholly different diseases. There was no danger in its performance, no neuritis, nor any neuralgic pain had ever followed it. It is supposed that the axis cylinder of the nerves are disintegrated, and the nerve fibers have been found in a granular condition after stretching. Paralysis has followed in some cases.

Dr. Fenger said it was not impossible that after a number of years the present operation might be looked upon as a surgical curiosity, but as it was useful in many cases, and harmless at most, it should be tried extensively.

### ELECTION OF OFFICERS.

Dr. H. M. Lyman, the founder of this society, was elected President, Dr. Mary G. Mergler Vice-President, and Dr. R. S. Hall was re-elected Secretary. Censors: Drs. Norman Bridge and E. L. Holmes.

